# Enhanced Oxidative Stress and Other Potential Biomarkers for Retinopathy in Type 2 Diabetics: Beneficial Effects of the Nutraceutic Supplements

**DOI:** 10.1155/2015/408180

**Published:** 2015-11-04

**Authors:** María J. Roig-Revert, Antonio Lleó-Pérez, Vicente Zanón-Moreno, Bárbara Vivar-Llopis, Juan Marín-Montiel, Rosa Dolz-Marco, Luis Alonso-Muñoz, Mara Albert-Fort, María I. López-Gálvez, David Galarreta-Mira, María F. García-Esparza, Carmen Galbis-Estrada, Carla Marco-Ramirez, Kian Shoaie-Nia, Silvia M. Sanz-González, Vicente Vila-Bou, Elena Bendala-Tufanisco, José J. García-Medina, Carlo Nucci, Roberto Gallego-Pinazo, J. Fernando Arévalo, Maria D. Pinazo-Durán, Valencia Study on Diabetic Retinopathy (VSDR)

**Affiliations:** ^1^Ophthalmic Research Unit “Santiago Grisolía”, Fundación Investigación Biomédica y Sanitaria (FISABIO), 90 Gaspar Aguilar Avenida, 46017 Valencia, Spain; ^2^Ophthalmology Research Unit, Department of Surgery, Faculty of Medicine and Odontology, University of Valencia, 15 Blasco Ibañez Avenida, 46010 Valencia, Spain; ^3^Ophthalmology Department, University Hospital “Arnau de Vilanova”, 12 San Clemente Street, 46015 Valencia, Spain; ^4^Ophthalmology Department, University and Polytechnic Hospital “La Fe”, 106 Fernando Abril Martorell Avenida, 46026 Valencia, Spain; ^5^Spanish Net of Ophthalmic Pathology OFTARED, Institute of Health Carlos III, 4 Sinesio Delgado Street, 28029 Madrid, Spain; ^6^Ophthalmology Clinic and Research Department, Clinica Oftalmológica Rahhal, 52 Cirilo Amorós Street, 46004 Valencia, Spain; ^7^Ophthalmology Department, Hospital Vinaroz, 1 Gil de Atrocillo Avenida, Vinaroz, 12500 Castellón, Spain; ^8^Ophthalmology Department, University Clinic Hospital, 1 Ramón y Cajal Street, 47005 Valladolid, Spain; ^9^Physiology Department, Faculty of Medicine, University Cardenal Herrera, 1 Seminario Avenida, Moncada, 46113 Valencia, Spain; ^10^Ophthalmology Department, University Hospital Reina Sofía, 1 Intendente Jorge Palacios Avenida, 30003 Murcia, Spain; ^11^Ophthalmic Unit, Department of Experimental Medicine and Surgery, University of Rome Tor Vergata, Rome, Italy; ^12^Retina Division, The Wilmer Eye Institute, The Johns Hopkins University School of Medicine, Baltimore, MD, USA

## Abstract

We have studied the global risk of retinopathy in a Mediterranean population of type 2 diabetes mellitus (T2DM) patients, according to clinical, biochemical, and lifestyle biomarkers. The effects of the oral supplementation containing antioxidants/omega 3 fatty acids (A/*ω*3) were also evaluated. Suitable participants were distributed into two main groups: (1) T2DMG (with retinopathy (+DR) or without retinopathy (−DR)) and (2) controls (CG). Participants were randomly assigned (+A/*ω*3) or not (−A/*ω*3) to the oral supplementation with a daily pill of Nutrof Omega (R) for 18 months. Data collected including demographics, anthropometrics, characteristics/lifestyle, ophthalmic examination (best corrected visual acuity, ocular fundus photographs, and retinal thickness as assessed by optical coherence tomography), and blood parameters (glucose, glycosylated hemoglobin, triglycerides, malondialdehyde, and total antioxidant capacity) were registered, integrated, and statistically processed by the SPSS 15.0 program. Finally, 208 participants (130 diabetics (68 +DR/62 −DR) and 78 controls) completed the follow-up. Blood analyses confirmed that the T2DMG+DR patients had significantly higher oxidative stress (*p* < 0.05), inflammatory (*p* < 0.05), and vascular (*p* < 0.001) risk markers than the T2DMG−DR and the CG. Furthermore, the A/*ω*3 oral supplementation positively changed the baseline parameters, presumptively by inducing metabolic activation and ameliorating the ocular health after 18 months of supplementation.

## 1. Introduction

Diabetes mellitus (DM), with a prevalence of logistic growth, rises pandemic proportions. It has been estimated that over 170 million people worldwide are currently affected by DM and it seems that these numbers will augment to over 360 million by 2030 [1]. A report on the incidence and prevalence of type 2 diabetes mellitus (T2DM) in 11 European countries showed that the age-adjusted/country-adjusted prevalence in 2004 was 10.2% in men and 8.5% in women and that these inequalities were highly related to the body mass index (BMI) [2]. In Spain, DM represents a big health problem, due to its prevalence of about 10% in the global population aged 30–89 years [3]. Number of diabetics among people aged 45 years or older in USA during 2012 was 24.6 million. Among them there were 2.1 million more diabetic men than women. The total (direct/indirect) estimated costs of DM in the same timeframe were $245 billion, and after adjusting for age/sex these data concluded that medical expenditures (in general) were double among diabetics compared with the no diabetics [4]. Other reports including large population collections also concluded that men had a higher T2DM prevalence than women [5, 6]. However, a more recent estimation of the risk factors for cardiovascular diseases described that diabetic women who smoke or are overweight develop cardiovascular conditions more frequently than men, with the same risk factors [7, 8].

Major diabetic complications are the result of a close interaction between genes and a wide variety of environmental factors. Diabetic retinopathy (DR), the microvascular complication of DM, is responsible for the decreased vision and blindness in young and middle-aged individuals. It has been estimated that about 80% of the type 2 diabetics (T2DM) and half of patients with type 1 DM (T1DM) develop DR in some point between the diagnosis and 15th year or more of the disease course [9, 10]. In this framework, it is well acknowledged that DR appear more frequently in T1DM than in T2DM patients and that DR prevalence end points positively correlate with the length of time afflicted by DM, as well as by the glycosilated hemoglobin (HbA1c), and hypertension blood pressure (HBP) levels [11, 12].

The retina is particularly sensitive to hyperglycemia. It has been shown that prolonged blood glucose elevation induces striking changes in oscillatory potentials in the electroretinogram (ERG), increased implicit times in the multifocal ERG (mtERG), and dyschromatopsia, whereas short-term hyperglycemia results in an overall decrease in the implicit times and increase in the amplitudes of the mtERG [13]. Chronic hyperglycemia induces retinal damage through different mechanisms, such as activation of protein kinase C, polyol, and hexosamine pathways, and/or generation of advanced glycation end products (AGEs). Nevertheless, hyperglycemia damages the endothelial cells and pericytes and increases vascular permeability with rupture of the blood-retinal barrier with the subsequent appearance of the retinal hypoxia processes, which, in turn, induces vascular endothelial growth factor (VEGF) expression, among other proangiogenic effectors [14, 15]. In this situation, independently of the presence or not of DR, main objectives for implementing eye care in diabetics have to include maximum nutritional and metabolic control.

Glycemic dysfunction has been widely associated with increased generation of reactive oxygen (O_2_) and nitrogen species (ROS, RNS) [16, 17]. These are continuously produced in cells during metabolic processes. Eventually, the electrons are transferred to molecular O_2_, by reducing this to H_2_O in a reaction catalyzed by the cytochrome c oxidase, accounting for almost 98% of the molecular O_2_ used by our body:

(1) $$\begin{array}{c} {\text{O}}_{2}+4{\text{e}}^{-}+4{\text{H}}^{+}⟶2{\text{H}}_{2}\text{O} \end{array}$$

It is known that 1-2% of the O_2_ undergoes an incomplete reduction by the mitochondria, generating the reactive oxygen species (ROS), very unstable forms such as the superoxide anion (O_2_^∙−^), formed when O_2_ is reduced by an electron. However, this molecule rapidly dismutates to form the hydrogen peroxide (H_2_O_2_) that can itself undergo dismutation by a reaction that is catalyzed by the manganese superoxide dismutase. The rate of production of O_2_/H_2_O_2_ depends on the Ca^2+^ availability. Importantly, the presence of transition metals, mainly iron, can induce H_2_O_2_ to generate the highly reactive hydroxyl radical OH^∙−^:

(2) $$\begin{array}{c} {\text{O}}_{2}+{\text{e}}^{-}={{\text{O}}_{2}}^{-}+{\text{H}}^{+}={\text{HO}}^{∙}+{\text{O}}_{2}⟶{\text{H}}_{2}{\text{O}}_{2} \end{array}$$

In fact, ROS/RNS originated from mitochondrial or nonmitochondrial sources have been related to DM, such as xanthine oxidase, NADPH oxidase, cyclooxygenase, lipoxygenase, cytochrome P450, and nitric oxide synthase. To counteract the effects of ROS/RNS, our body depends on the endogenous and exogenous antioxidants (AOX), including the enzymes superoxide dismutase, catalase, glutathione peroxidase, and glutathione reductase [18].

Nevertheless, ROS overproduction or its deficient removal leads to vascular disorders that result in protein fragmentation, amino acids aggregation, membrane lipids alteration, and/or nucleic acids injury. These described changes may trigger cell damage and death. From the described oxidative background important biomarkers can emerge, and obviously these molecules can be determined in DM patients, especially in those suffering chronic diabetes-related complications [19, 20].

The retina is continuously exposed to light. Therefore retinal cells live under chronic oxidative stress conditions, also induced by the high O_2_ requirements and the high O_2_ partial pressure from the underlying vessels of the choriocapillaris. It has also to be considered that the photoreceptor outer segments are very rich in polyunsaturated fatty acids (PUFAs), mainly the omega 3 docosahexaenoic acids (DHA), which are extremely susceptible to lipid peroxidation and protein modifications that may be induced by the lipid peroxidation by-products [21]. Current knowledge on the physiological and pathological mechanisms of oxidative stress in DR has been collected from a wide spectrum of both the animal models research and the epidemiological studies [22, 23]. However, clinical trials have provided ambiguous results on the effects of the antioxidant [24, 25] and PUFAs [26, 27] supplements on the development and progression of DR.

Chronically elevated glucose/HbA1c levels altogether with a series of endogenous and exogenous risk factors lead to the retinopathy. The primary goal of the present study is to investigate the risk of retinopathy in a Mediterranean population of T2DM patients, according to clinical, biochemical, and lifestyle biomarkers, the oxidative stress status being among them. Secondary goal was to evaluate the effects of the oral administration of supplements with antioxidants and omega 3 fatty acids (A/*ω*3) in the oxidative stress outcomes and the inflammation and vascular risk biomarkers, to better manage eye care and vision among diabetics.

## 2. Material and Methods

### 2.1. Community-Based Study Design

The study was done in agreement with the Declaration of Helsinki for human studies (as revised in Edinburgh, 2000), and all records were treated according to the Spanish law for protecting personal data (LOPD: Organic Law 15/1999, de 13 Dic). Our study was validated by the Ethics Committee and Clinical Research of the University Hospital Dr. Peset of Valencia (Spain), the main center of the Research Project (number 12/33), and by the Ethics Committees of all participating institutions. Moreover, the study received the permission of the Agencia Española de Medicamentos y Productos Sanitarios (AEMPS).

We conducted a prospective multicenter study lasting for 18 months (Jan 2013–June 2014) that was carried out in 10 centers by 32 investigators, with the main goal of delineating the natural history of DM and eye disease as well as the risk factors for DR in T2DM patients and to evaluate whether A/*ω*3 supplements could increase compliance with the health and vision care among diabetics.

The participant investigators were selected to obtain stratified ophthalmologists from main hospitals pertaining to the National Health System in the Valencia Community and ophthalmology clinics, as well as basic researchers involved in ophthalmology and vision sciences.

**Figure 1:**
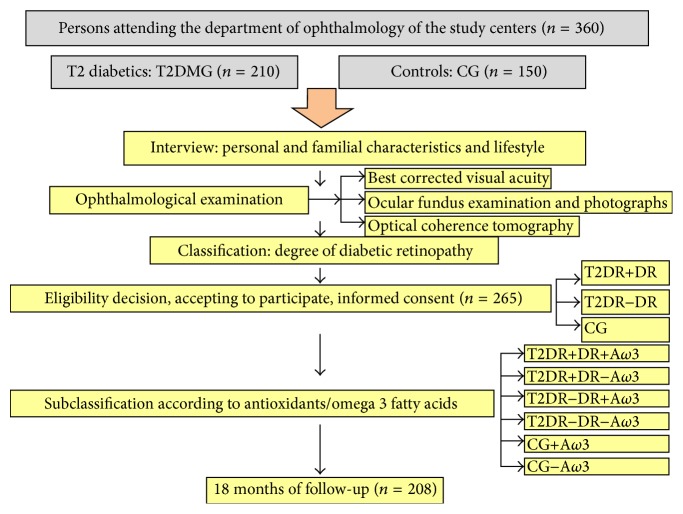
Flowchart of the challenges for the recruitment and screening methods of the study participants: characteristics of recruitment, sample size, and study design.

A total of 360 persons (25–80 years old) of both sexes were preinterviewed for eligibility for the study that was determined by the main investigator, according to the inclusion/exclusion criteria listed in Table 1. First point for recruitment was the diagnosis of T2DM and the duration of disease since diagnosis. In parallel, healthy individuals were also recruited. Therefore, from the initial sample, 265 suitable participants were enrolled, signed the informed consent, and were appointed to the first visit of this interventional study. Then, office visits were programmed every six months, including periodical telephone calls from the investigators throughout the study. Total study duration was 18 months and the characteristics of recruitment are enclosed in Figure 1.

### 2.2. Patients and Methods

#### 2.2.1. Interview

Participants who completed the first enrollment interview (*n* = 265) were enclosed for demographics, patient characteristics, and lifestyle. The following variables were recorded: age, sex, ethnicity, DM duration, BMI (height, weight), familial background, current medication, smoking/drinking habits, and physical exercise. In the mid-twentieth century, Professor Ancel Keys observed the nutritional habits of people living in the European Mediterranean countries leading to the conclusion that a significant reduction in the incidence of chronic diseases and a higher lifespan were main characteristics of this area (*for further reading, Keys et al. The diet and 15-year death ratein the seven countries study. Am J Epidemiol. 1986; 124:903*). Major details of this type of diet are (1) the elevated consumption of fruits, vegetables, legumes, olive oil, bread, and cereals, (2) the moderate-to-high ingestion of fish and chicken meat, (3) the moderate intake of cheese, yogurt, and wine, and (4) the low levels of consumption of red meat. The UNESCO raised the Mediterranean diet in 2013 as a lifestyle in itself and a cultural heritage for the humanity. We used a 14-item questionnaire to assess the adherence of the sample participants with the Mediterranean diet (AMD). The Mediterranean diet scores indicating compliance to the Mediterranean diet distinguished between the participants with a high intake of cereals, legumes, fruits, vegetables, olive oil, fish, bread, and red wine that were scored positive (1), while those with a low intake of these meals were scored negative (0). The results of this questionnaire were reflected as participants having either good adherence to the Mediterranean diet (GAMD) or poor adherence to the Mediterranean diet (PAMD).

The T2DMG (*n* = 165) was subclassified according to having or not having DR. Furthermore, each of these subgroups was homogeneously subdivided into those participants that were randomly assigned to take or not one pill per day of the A/*ω*3 supplementation (T2DMG+DR+A/*ω*3/T2DMG+DR−A/*ω*3; and T2DMG–DR+A/*ω*3/T2DMG–DR−A/*ω*3). The CG (*n* = 100) was also randomly assigned to those taking (CG+A/*ω*3) or not (CG−A/*ω*3) the A/*ω*3 supplements. A flow chart with the number of patients pertaining to the groups and subgroups is enclosed in Figure 1. The nutraceutic formulation contains docosahexaenoic acid (DHA), vitamins (E, C, B1, B2, B3, B6, B9, and B12), lutein, zeaxanthin, glutathione, hydroxytyrosol, and trace elements (Se, Mn, Zn, and Cu), commercialized as Nutrof Omega, commercialized by Laboratorios Thea SA (Barcelona, Spain) that gently provided all the oral supplementation needed for the study participants. The study-related visits and the nutraceutic supplements (Nutrof Omega) were given to our participating volunteers (randomly chosen) without any charge.

#### 2.2.2. Ophthalmic Examination

A systematized ophthalmological examination was performed to our 265 participants including best corrected visual acuity (BCVA) with each eye, ocular fundus examination and retinographs (ImageNet; Topcon, Barcelona, Spain), and optic coherence tomography (OCT) examination (Spectral Domain SD-OCT; Zeiss, Madrid, Spain).

The DR diagnosis was done from ocular fundus examination and photographs which were carried out in each eye of the T2DG (*n* = 165), based on the Early Treatment of Diabetic Retinopathy Study (ETDRS), and the DR severity was graded as described before [28]. The presence and number of microaneurysms, hemorrhages, venous beading, and/or intraretinal microvascular abnormalities were considered the most relevant factors for monitoring DR progression. In fact, retinopathy worsened basically by overall augment in counts of microaneurysms and haemorrhages. The ETDRS retinopathy severity scale divides the process into 13 levels ranging from absence of retinopathy to severe vitreous hemorrhage.

The OCT parameters were measured with the Cirrus SD-OCT direct cross-sectional imaging device of Zeiss Meditec (Dublin, CA, USA) in a total of 265 eyes with the clinical diagnosis of nonproliferative diabetic retinopathy (T2DG; (*n* = 165) with or without DR and/or clinically significant macular edema) and in the healthy participants (CG; *n* = 100). Prior to the OCT examination, each participant was administered a drop of tropicamide in both eyes to dilate the pupils. Each ophthalmologist visualized the representative A-scan and manually moved the measurement cursors (first at the signal that represents the internal limiting membrane and second at the signal that denotes the retinal pigment epithelium). Then, the most profound zone of the foveal pit was taken as the center. Six consecutive scans were performed for each eye. Cross-sectional retinal images were optimized for each scan to obtain the highest intensity/definition; therefore these scans with signal strengths ≥ 7 and without artifacts were included in this study. Briefly, acquisition protocols were macular cube 200 × 200; HD 5 line raster/5 raster line; and HD one line. The raw OCT datasets were exported to a personal computer for analysis. The SD-OCT system analyzed retinal thickness, creating a topographic map and graphs for quantitatively and qualitatively documenting any changes in retinal thickness and edema. Main exclusion criteria for this technique were any retinal pathology other than DR or opaque media such as in cataracts and inability to undergo the OCT examination due to mobility or cognitive difficulties.

#### 2.2.3. Sample Processing

At baseline and every 6 months of the follow-up, all participants were visited in the corresponding centers and the peripheral blood was collected from the antecubital vein under fasting conditions (between 8:00 and 9:00 a.m.). Part of the obtained blood tubes was analyzed by computerized systems to determine laboratory blood draws and analysis: glucose, glycosylated hemoglobin (HbA1c), cholesterol (HDL, LDL), and triglycerides. Two more EDTA tubes were obtained and transported in optimal conditions for preservation to the ophthalmic research centers. Each blood sample was centrifuged at 3000 rpm for 10 min and the plasma and erythrocytes were separated into Eppendorfs, labeled, and registered to be stored at −80°C until processing, as described below.

*(1) Determination of Malondialdehyde- (MDA-) Thiobarbituric Acid Reactive Substances (TBARS).* Thiobarbituric acid reactive substances (TBARS) including MDA were measured in plasma samples, as lipid peroxidation by-products. Briefly, at high temperature (90–100°C) under acidic conditions MDA-TBA complex was formed by the reaction between these molecules and extracted with butanol, according to previous descriptions [28, 29]. The fluorescence of these complexes was measured in duplicate at 544 nm excitation and 590 nm emission deep, relative to the fluorescence of the standard samples, and then the concentration of the samples was calculated by extrapolating the results in the standard curve. 

*(2) Total Antioxidant Activity (TAA)*. The antioxidant activity was measured in the plasma samples by means of the antioxidant assay (Cayman Chemical Company, Ann Arbor, MI) commercial kit. The kit is based on the antioxidant capacity of plasma to inhibit the oxidation of ABTS (2,2′-azino-di-[3-ethylbenzthiazoline sulphonate]) to ABTS^+^ by the metmyoglobin, as previously reported [30, 31].

### 2.3. Statistical Procedures

For statistical analysis, the excel program and the SPSS 15.0 (SPSS Inc., Chicago, IL, USA) were used. All the programmed experiments were performed in duplicate for each sample. The continuous variables were expressed as mean ± standard deviation of the mean (SD). Categorical variables were expressed as percentages. The statistical test performed utilized the two/tailed test at 5% level of significance. Appropriate analysis of covariance models was also performed.

## 3. Results

From the initial suitable participants (*n* = 265), a total of 208 participants (130 T2DM patients (62 +DR versus 68 −DR) and 78 healthy controls) continued and completed altogether the 18 months of the study.

Demographics, patient characteristics, and lifestyle details of the study participants are listed in Table 2. It has to be emphasized that the HBP was twice frequent in the T2DMG+DR than in the diabetics −DR (*p* < 0.001).

The BCVA (each eye in separate) was lower in the T2DMG than in the CG [(CG at baseline: RE: 0.95 ± 0.10 and LE: 0.96 ± 0.08; *p* < 0.001/CG; at 18 months: RE: 0.95 ± 0.11 and LE: 0.95 ± 0.10; *p* < 0.001) (T2DMG at baseline: RE: 0.81 ± 0.30 and LE: 0.83 ± 0.22; *p* < 0.001/T2DMG; at 18 months: RE: 0.78 ± 0.22 and LE: 0.76 ± 0.22; *p* < 0.001)].

**Figure 2:**
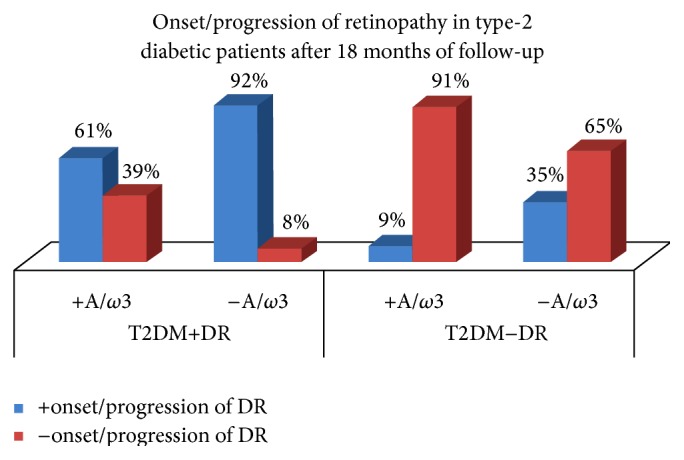
Percentages of development or progression of DR in the participants during the study course, according to being assigned or not to the oral supplementation. +A/*ω*3: oral supplementation with antioxidants and omega 3 fatty acids. –A/*ω*3: without taking the oral supplementation. T2DM+DR: type 2 diabetics with retinopathy. T2DM−DR: type 2 diabetics without retinopathy.

The baseline ocular fundus examination revealed that 62 patients of the T2DMG displayed DR with the following degrees of severity: 63.6%: mild DR, 29.1%: moderate DR, and 7.3%: severe DR. From baseline to the end of study, 11 patients remained without signs of DR progression, but the rest progressed as follows: 29.1% displayed mild DR, 36.4% moderate DR, and 14.5% severe DR. Furthermore, the 68 T2DMG patients that did not have DR showed progression of disease at the end of study in the following proportions: 14.3% of the patients without DR progressed to mild DR, 7.1% of the patients without DR progressed to moderate DR, and no progression to severe degree of DR was detected through the follow-up in any of these patients. In our study, the T2DMG displayed DME (clinically significant) at baseline 5.6% DME in the RE versus the 11.2% in the LE (*p* = 0.037, *p* = 0.003, resp.). Moreover, DME was more frequent in the T2DMG+DR than in the T2DMG−DR and the CG (*p* = 7.036^*E*−005^ for the RE and *p* = 2.214^*E*−006^ for the LE). At the end of study, it was seen that DME was significantly more frequent in patients with severe DR (50%) than in those with mild or moderate DME (*p* = 0.03). A summary of the progression characteristics based on the ocular fundus and SD-OCT examination of the study participants is reflected in Figure 2.

All the results obtained from the ophthalmic examination of the study participants are enclosed in Table 3.

Classical and oxidative stress hematologic parameters from the T2DM participants are enclosed in Table 4. Parameters related to oxidative stress (lipid peroxidation (MDA/TBARS), total antioxidant activity (TAA)) showed that the plasmatic MDA/TBARS levels were significantly higher, and the TAC was significantly lower in diabetics as compared to the CG (Table 4). It was also observed that the MDA/TBARS displayed significantly higher concentrations and the TAA showed significantly lower activities in the T2DMG+DR with respect to those diabetics without DR and the CG (Table 4). Data from the oxidative stress parameters according to the degree of severity of DR (ETDRS international scale) are reflected in Table 5. The MDA/TBARS levels augmented with DR progression, but the TAA significantly decreased with DR progression.

The adherence to the Mediterranean diet nutritional facts of the study participants were reflected in the 14-item questionnaire. Specifically, average values for the 14-item score were significantly higher in the CG (9.8 ± 2.0) versus the T2DMG (6.4 ± 1) (*p* < 0.05). The comparison of the prooxidant and antioxidant markers analyzed in the present study in the T2DMG with poor and good adherence to the Mediterranean diet (PAMD/GAMD) is reflected in Table 6.

**Figure 3:**
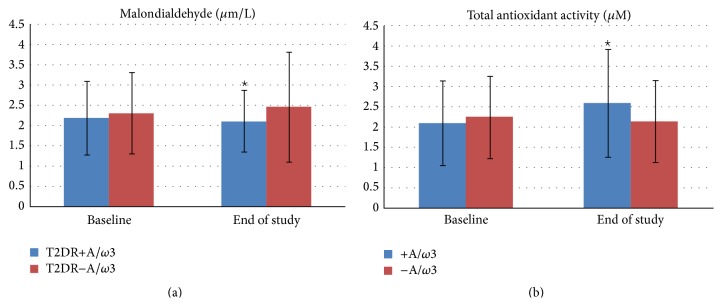
Oxidative and antioxidant status in the type 2 diabetics during the 18 months of follow-up, according to being assigned or not to the oral supplementation of A/*ω*3 supplements. T2DR+A/*ω*3: type 2 diabetics with retinopathy taking the oral supplementation; T2DR−A/*ω*3: type 2 diabetics with retinopathy not taking the oral supplementation; +A/*ω*3: assigned to the oral supplementation; −A/*ω*3: not assigned to the oral supplementation.

When assessing the oxidative stress status at the end of the eighteen months of the follow-up, it was seen that plasmatic MDA/TBARS levels significantly decreased in the T2DM+A/*ω*3 subgroup with respect to the T2DM−A/*ω*3 patients (Figure 3(a)). Furthermore, the TAC significantly increased in the T2DM+A/*ω*3 subgroup, while the nonsupplemented participants did not show any noticeable change (Figure 3(b)).

## 4. Discussion

The retinopathy is the major cause of blindness among adult diabetics (aged 20–75 years) in the developed world [1–6]. In this population-based multicenter study carried out in 265 participants from the Mediterranean area of Spain (T2DMG; *n* = 165 versus CG; *n* = 100), “*The Valencia Study on Diabetic Retinopathy (VSDR)*,” we have identified significant risk factors of DR, such as the HBP, duration of the DM, physical exercise, the BMI, the smoking/drinking habits, the dyslipidemia, and the oxidative stress. In fact, plasmatic oxidative stress biomarkers increased and antioxidant activity decreased in the diabetics with DR, with respect to those without DR and the healthy controls, data consistent with previous reports from our group and other authors [18, 19, 22–25]. More precise information about each of these risk factors is provided below.

The HBP was present in 76.4% of our T2DM+DR versus 58.6% of the T2DM−DR and 9.3% of the CG (*p* < 0.001). Emdin et al. [32] studying patients with T2DM emphasized that appropriated HBP lowering was related to improved mortality and other positive clinical changes in the affected patients. Barrios and Escobar [33] reported that DM and HBP are closely related disorders creating an optimum background for atherosclerosis. In fact, the strict control of both processes is pivotal to ameliorate macroangiopathy/microangiopathy prognosis in the affected patients.

The lead time between the diagnosis of the disease and the first appointment for the present study was considered as the duration of DM. Average DM durability in our participants was 14.2 ± 8.2 years. Furthermore, in the T2DMG+DR, it was 19.2 ± 6.8 years while in those without retinopathy the duration of DM was 10.4 ± 7.1 years. In our work this parameter was also related to the degree of severity of DR, as shown in Table 5. We demonstrated that the duration of DM was strongly associated with a higher risk of retinopathy (OR = 1.18 *p* < 0.001). Other authors also reported a strong relationship between DM duration and retinopathy, neuropathy, nephropathy, and peripheral vasculopathy in a sample of 1,157 individuals of Northwest India [34].

Healthy participants performing physical exercise accounted for almost double than the diabetics (CG: 68% versus T2DMG 37.6%; *p* < 0.001). Regarding the presence of retinopathy, significantly higher percentage of subjects performing physical exercise was observed in T2DMG−DR than in the T2DMG+DR (45% versus 27%; *p* = 0.035). Our results coincide with those reporting that physical exercise is beneficial with respect to both the glycemic control and the diabetes-related comorbidities, including the vitreoretinal disorders [35].

The importance of data regarding the BMI from our participants was notorious. Significant differences between the CG and the T2DMG (13.8 + 3.8 kg/m^2^ versus 28.4 + 4.3 kg/m^2^; *p* < 0.001) were detected. When the presence of retinopathy was analyzed, it was seen that the differences of BMI between the T2DMG+DR and T2DMG−DR were not significant (28.8 + 4.7 kg/m^2^ versus 28.0 + 4.0 kg/m^2^; *p* = 0.345). In agreement with these data, it has been reported that BMI in correlation with HbA1c, HBP, and cholesterol seems to be related to DR progression in T2DM patients [36]. Furthermore, it has been suggested that BMI may be considered as a predictive marker for the development and progression of DR [36, 37]. In addition to the carbohydrate restriction, dietary recommendations on this topic include saturated fat restriction (<7% of daily caloric intake) and cholesterol restriction (<200 mg/dL) among diabetics.

Tobacco use is a major cause of disease, disability, and premature death worldwide. Our results showed a higher frequency of smokers among the T2DMG than in the CG (34.4% versus 20%, resp.; *p* = 0.03). Diabetics also displayed higher alcohol consumption than the healthy participants (24.8% versus 17.3%) but the difference was not statistically significant (*p* = 0.217). Furthermore, the T2DMG+DR showed significant differences regarding tobacco and alcohol consumption with respect to the T2DMG−DR (*p* = 0.001 and *p* < 0.001, resp.). In fact, it has been described that drinking in excess has to be avoided because it induces ketoacidosis and hypertriglyceridemia. Moreover, it has to be emphasized that alcohol taken outside the meals can cause hypoglycemia crisis and a relevant increase of a host of health problems [38, 39].

Oxidative stress, affecting the cell and tissue morphology and functions, as well as leading to cell death, is a major player in the pathogenesis of a wide variety of retinal diseases. This fact called our attention regarding DR, because knowledge on the precise molecular mechanisms of DR development and progression is far from complete. Up to date, there is no definitive treatment for the disease. In the Mediterranean Spanish region of Valencia, 500.000 diabetics (from a total of 5.004.844 inhabitants) have been recorded in the last year. Blood samples from our diabetics were analyzed for the oxidative and antioxidant activities, as well as for determining the glycemia, HbA1, HDL/LDL cholesterol, and triglycerides. Despite the classical parameters that were significantly higher in diabetics (glycemia, HbA1C), as expected, it was quite surprising that the triglycerides levels were significantly lower in the CG than in the T2DMG at baseline (97.5 ± 59.8 mg/dL versus 149.6 ± 96.6 mg/dL; *p* < 0.001) and after 18 months (114.7 ± 69.3 mg/dL versus 142.6 ± 80.9 mg/dL; *p* = 0.014). However, this data was not statistically significant between the diabetics with/without DR at baseline (133.63 ± 65.16 mg/dL versus 156.86 ± 114.85 mg/dL; *p* = 0.385) neither after 18 months (134.0 ± 65.9 mg/dL versus 141.3 ± 91.0 mg/dL; *p* = 0.812). Other authors concluded that no significant association between DR, triglycerides, HDL, and total cholesterol was detected in a sample of Danish diabetics [40]. Miljanovic et al. did not find associations between lipid profile and DR progression [41]. In contrast, the global case-control study in 13 countries concluded that DR was associated with triglycerides and high-density lipoprotein cholesterol in matched analysis [42].

It has been widely recognized that MDA/TBARS are associated with the production of AGEs implicated in the DR development. This mechanism is mainly due to AGEs accumulation in the ocular tissues by increasing cell permeability, angiogenesis, and disruption of the internal blood-retinal barrier [43]. Plasmatic MDA/TBARS levels were significantly higher and the TAA levels were significantly lower in the T2DMG than in the CG. Furthermore, the oxidative status was significantly higher and the antioxidant activity was significantly lower in diabetics +RD with respect to diabetics −RD, data similar to previous reports demonstrating enhanced oxidative stress in T2DM patients [16–19, 23, 28]. Interesting results came from the relationship between the DR degree of severity and the plasmatic oxidative stress markers, pointing to the fact that the prooxidants significantly increased and the antioxidants significantly decreased in plasma samples of the T2DMG according to the ETDRS severity scale, as shown in Table 5. Data strongly suggests that the hyperglycemia created a particular oxidative stress background in the retina of the participant diabetics that, in turns, influences the onset and progression of DR, as reflected throughout the 18 months of the follow-up.

We also observed that a nutraceutic formulation containing A/*ω*3 is capable of counteracting the oxidative stress detected in this diabetic Mediterranean population, as reflected in Figure 2 and Tables 4–6. In addition to the antioxidant enzymes, the robust antioxidant capacities of a high variety of nonenzymatic antioxidants—among them are vitamins (ascorbic acid (vit C), *α*-tocopherol (vit E)), transition metals (selenium (Se), zinc (Zn), copper (Cu), and manganese (Mn)), glutathione, carotenoids (*β*-carotene, lutein, and lycopene), hormones (melatonin, oestrogen), phenols (catechin, quercetin), and so forth—and the added benefits of the antioxidant and anti-inflammatory properties of the PUFAs are well defined in the literature [21, 22, 24–27]. Therefore, possible protecting mechanisms against oxidative stress of the oral supplementation with a combination of A/*ω*3 in our diabetics may include a mixture of the strong antioxidant activity from vitamins C and E, the carotenoids lutein and zeaxanthin, the free radical scavenging glutathione, and the trace elements Se, Zn, Cu, and Mn, as well as the DHA and DHA-metabolic by-products, all of which are participating in scavenging excessive oxidants, preventing free radical generation, recycling other antioxidants, and probably ameliorating the mitochondrial functions (oxidative phosphorylation). In this framework, it has been reported that systemic interventions are highly required for controlling DR patients. Among these, early detection of hyperglycemia and intensive glycemic control together with hypertension and dyslipidemia therapy have to be programmed. As a consequence of the results raised during this study course, strategies for avoiding DR development and progression should therefore aim to prevent specifically the effects of glycation but also the oxidative stress consequences. Therefore, it is reasonable to consider that biotherapies with multiple targets can be more useful than unique drugs in DR treatment [43].

However, it is notorious that studies with oral supplements on DR have yielded conflicting findings. Among the studies on the role of A/*ω*3 in the prevention of DR, the San Luis Valley Diabetes Study did not find protective effect in DR and vitamin E on DR of the b-carotene, vitamin C, and vitamin E [44]. The Third National Health and Nutrition Examination study described that *α*-tocopherol and vitamin C serum levels were not associated with DR [45]. Moreover, the Diabetes Control and Complications Trial described that patients assigned to a low-fat diet decreased the rate of DR progression as compared with patients not following the low-fat diet [46]. In addition, antioxidant and antiangiogenic properties of edible berries were shown to be protective for DR [47]. García-Medina et al. [48] designed a follow-up study in the Mediterranean area of Spain in T2DM patients with nonproliferative diabetic retinopathy, evaluating the effect of A/*ω*3 supplementation over a 5-year follow-up. The DR stage showed a retardation of progression in the subgroup with supplementation but worsened in the nonsupplemented subgroup. It was also described that the oral supplements maintained the antioxidant defenses activity as shown by the high plasmatic TAA levels, which was related to the decreased oxidative plasma activity. In several experimental studies using different strains, it was shown that increasing the diversity of antioxidants provides significantly more protection than using a single antioxidant for the supplementation. Moreover, when antioxidants were administered to diabetic rats with the main goal of assessing the ability of these agents to inhibit the development of DR, the authors concluded that long-term administration of antioxidants could inhibit the development of the early phases of DR [49]. Hence, the oral supplementation with A/*ω*3 represents an achievable adjunct to help preserve vision in diabetics.

It is recognized that proliferative diabetic retinopathy (PDR) is the most usual sight-threatening vitreoretinal damage in T1DM patients, while diabetic macular edema (DME) is considered the main cause of visual impairment and blindness in the T2DM patients [50, 51]. In our study, the T2DMG displayed DME (clinically significant) at baseline 5.6% DME in the RE versus the 11.2% in the LE (*p* = 0.037, *p* = 0.003, resp.). Moreover, DME was more frequent in the T2DMG+DR than in the T2DMG−DR and the CG (*p* = 7.036^*E*−005^ for the RE, and *p* = 2.214^*E*−006^ for the LE). Among the recognized risk factors are the hyperglycemia, HBP, dyslipidemia, DM duration, and oxidative stress status. However, our study supports the benefits of the A/*ω*3 supplements as well as the important modifiable component for the risk factors profile for better managing the diabetic eyes and vision.

One additional interesting aspect of our work is that compliance with the Mediterranean diet was assessed by a brief 14-item validated questionnaire which is faster and less complicated to the participants than other questionnaires, being considered very useful by the investigators [52, 53]. The Mediterranean diet is a nutritional and lifestyle pattern based on the traditional dietary habits of Greece, Italy, and Spain [52]. The term Mediterranean diet was set by Ancel Keys in 1960. In our study course, we have detected that the average values of the 14-item Mediterranean diet scores were significantly higher in the CG versus the T2DMG (*p* < 0.05). Moreover, comparison of the prooxidant and antioxidant markers in the T2DMG with GAMD or PAMD also reported significant differences (Table 6). These results point to those people with GAMD with the above described dietary habits who had a lesser risk of DR, which agree with previous reports regarding the lesser risk of chronic and cardiovascular diseases among the Mediterranean countries inhabitants [50–52]. At this point, we may suggest that this type of questionnaire is useful for evaluating the role of the Mediterranean diet in the ocular diseases.

A limitation of this study was the relatively small sample size of 265 participants at baseline (T2DMG; *n* = 165 versus CG; *n* = 100) and the important patients lost to follow-up (a total of 208 participants—among them are 130 patients from the T2DMG (62 +DR versus 68 −DR) and 78 healthy individuals from the CG—continued and completed altogether the 18 months of the study). For this reason, these findings are helpful enough to understand the characteristics of the studied population, but we are trying to control these important variables for further research, to better generalize our results to the broader community.

## 5. Conclusions

Healthy living includes everything we have to plan for improving our wellbeing. Specifically, diabetics have to be able to handle life's challenges, including exercise, being aware of smoking and drinking in excess, and maintaining the body weight. All of these have to be considered key milestones for eye care in diabetics. The DM induced an oxidative background in T2DM participants of the Mediterranean study area that was enhanced in patients +DR. The A/*ω*3 formulation utilized herein provided the appropriate vitamins, minerals, and PUFAs, resulting in decreased plasmatic oxidative level and increased antioxidant activity in diabetics. Antioxidant supplementation may be useful to help manage diabetics at risk of retinopathy and vision loss.

## Figures and Tables

**Table 1:** Inclusion and exclusion criteria for the study participants. Criteria for eligibility in the Valencia Study on Diabetic Retinopathy (VSDR).

Inclusion	Males and females, aged >25 years and <80 years with type 2 diabetes for 12 months (at least)
	Insulin naïve
	Healthy individuals as controls
	No ocular or systemic diseases or aggressive treatments and no ocular surgery or laser for 12 months (at least); no other oral supplements with antioxidants and/or omega 3 fatty acids
	Provided written informed consent before any related activities commence
	Participants able to attend the visits and to follow the study guidelines

Exclusion	Males and females, aged <25 years and >80 years
	Insulin dependent patients
	Ocular or systemic diseases or aggressive treatments and/or previous ocular surgery or laser
	Antioxidant and/or omega 3 fatty acids supplements
	No acceptance for the study participation and/or not signing the informed consent
	Participants unable to attend the visits or to follow the study guidelines

**Table 2:** Sociodemographic characteristics of diabetics with/without retinopathy.

	T2DM+DR	T2DM−DR	*p* ^*∗*^
Age (years)	65.1 ± 8.6	62.3 ± 10.1	0.094
Gender (% of women)	52.7	48.6	0.645
DM duration (years)	19.2 ± 6.8	10.4 ± 7.1	<0.001^*∗*^
Physical exercise (%)	27.3	45.7	0.035^*∗*^
Smoking (%)	43.6	15.7	0.002^*∗*^
Alcohol drinking (%)	38.2	8.6	<0.001^*∗*^
BMI (kg/m^2^)	28.8 ± 4.6	28.0 ± 4.0	0.345
GAMD (%)	18.2	44.3	0.002^*∗*^

T2DM+DR: type 2 diabetes mellitus with diabetic retinopathy; T2DM−DR: type 2 diabetes mellitus without diabetic retinopathy; DM: diabetes mellitus; BMI: body mass index; GAMD: good adherence to Mediterranean diet. Data are shown as mean ± standard deviation.

^*∗*^Statistically significant (*p* < 0.05).

**Table 3:** Ophthalmic examination records of the study participants.

		T2DM
BCVA RE	Baseline	0.81 ± 0.296
	18 months	0.78 ± 0.220
	*p* value	0.176

BCVA LE	Baseline	0.83 ± 0.22
	18 months	0.78 ± 0.22
	*p* value	0.388

IOP RE (mmHg)	Baseline	15.2 ± 2.8
	18 months	15.6 ± 2.5
	*p* value	0.099

IOP LE (mmHg)	Baseline	15.6 ± 2.9
	18 months	16.1± 2.5
	*p* value	0.048^*∗*^

RNFLT RE (*μ*m)	Baseline	251.65 ± 22.79
	18 months	254.14 ± 31.60
	*p* value	0.001^*∗*^

RNFLT LE (*μ*m)	Baseline	258.53 ± 55.029
	18 months	262.22 ± 6.38
	*p* value	0.011^*∗*^

MT RE (*n*)	Baseline	5.6% (13)
	18 months	7.2% (9)

MT LE (*n*)	Baseline	11.2% (14)
	18 months	10.4% (13)

DM − R: diabetes mellitus without retinopathy; DM + R: diabetes mellitus with retinopathy; BCVA: best corrected visual acuity; RE: right eye; LE: left eye; IOP: intraocular pressure; RNFL: retinal nerve fiber layer thickness; MT: macular thickness. Data are shown as mean ± standard deviation.

^*∗*^Statistically significant (*p* < 0.05).

**Table 4:** Haematologic parameters in diabetics with/without retinopathy at baseline and at 18 months of follow-up.

	T2DM−DR		*p*	T2DM+DR		*p*
	Baseline	End of study		Baseline	End of study	
MDA (*µ*m/L)	2.37 ± 1.39	2.43 ± 0.98	0.724	3.63 ± 1.30	3.83 ± 1.81	0.482
TAS (*µ*M)	2.64 ± 1.52	2.87 ± 1.31	0.203	1.96 ± 1.32	2.09 ± 1.39	0.020^*∗*^
HbA1c (%)	7.12 ± 1.55	6.99 ± 1.30	0.307	7.87 ± 1.48	8.72 ± 1.45	0.046^*∗*^
Total-c (mg/mL)	186.8 ± 39.4	184.3 ± 37.6	0.669	178.8 ± 32.6	181.3 ± 33.8	0.522
HDL-c (mg/mL)	50.4 ± 13.1	51.6 ± 14.2	0.479	51.5 ± 13.0	51.4 ± 13.0	0.959
LDL-c (mg/mL)	104.3 ± 28.6	102.3 ± 35.2	0.687	99.9 ± 29.4	102.6 ± 35.4	0.587

DM − R: diabetes mellitus without retinopathy; DM + R: diabetes mellitus with retinopathy; MDA: malondialdehyde; TAS: total antioxidant status; HbA1c: glycosilated hemoglobin; total-c: total-cholesterol; HDL-c: higher density lipoprotein-cholesterol; LDL-c: lower density lipoprotein-cholesterol. Data are shown as mean ± standard deviation.

^*∗*^Statistically significant (*p* < 0.05).

**Table 5:** Oxidative status in diabetics according to the degree of retinopathy (*ETDRS International scale*).

T2DMG	MDA/TBARS (*µ*mol/L)	TAA (*µ*mol/L)
−DR	1.19 ± 0.53	3.84 ± 1.24
+DR		
Mild DR	3.00 ± 1.74	2.38 ± 1.30
Moderate DR	4.94 ± 1.29	1.29 ± 0.59
Severe DR	5.36 ± 0.51	1.10 ± 0.34
*p* value^*∗*^	2.997*E* − 006^*∗∗*^	4.857*E* − 005^*∗∗*^

−DR: diabetes mellitus without retinopathy; +DR: diabetes mellitus with retinopathy; MDA: malondialdehyde; TAA: total antioxidant activity. Data are shown as mean ± standard deviation.

^*∗*^ *p* value obtained from ANOVA analysis.

^*∗∗*^Statistically significant (*p* < 0.05).

**Table 6:** Comparison of the plasmatic prooxidant and antioxidant markers found in the diabetes mellitus patients according to having poor/good adherence to Mediterranean diet.

	T2DM−DR		T2DM+DR		*p* ^*∗*^
	PAMD	GAMD	PAMD	GAMD	
MDA1 (*µ*m/L)	2.30 ± 1.14	2.47 ± 1.67	3.77 ± 1.26	3.01 ± 1.32	<0.001^a,b^
MDA2 (*µ*m/L)	2.32 ± 0.85	2.58 ± 1.11	4.12 ± 1.78	2.49 ± 1.31	<0.001^a,b,c^
TAA1 (*µ*M)	2.57 ± 1.50	2.71 ± 1.58	1.86 ± 1.10	2.39 ± 2.06	0.051
TAA2 (*µ*M)	3.11 ± 1.50	2.58 ± 0.99	1.91 ± 1.38	2.88 ± 1.17	0.001^a^

T2DM−RD: type 2 Diabetes mellitus patients without diabetic retinopathy; T2DM+DR: type 2 diabetes mellitus patients with diabetic retinopathy; PAMD: poor adherence to Mediterranean diet; GAMD: good adherence to Mediterranean diet; MDA1: malondialdehyde-baseline; MDA2: malondialdehyde-end of study; TAA1: total antioxidant activity-baseline; TAA2: total antioxidant activity-end of study. Data are shown as mean ± standard deviation.

^*∗*^ *p* value obtained from ANOVA analysis.

a: significant differences between DM − DR PAMD and DM + DR PAMD; b: significant differences between DM − DR GAMD and DM + DR PAMD; c: significant differences between DM + DR PAMD and DM + DR GAMD.
